# Reminiscences of the Insane

**Published:** 1886-12-04

**Authors:** 


### Reminiscences of the Insane.

By a Mental Physician.

The Public and Plausible Patients.

It sometimes happens that persons outside asylums are carried away by the strong protest and plausible statements of patients into a belief of their sanity, and take up their cause with a view to obtain their liberation, when those who are familiar with the true nature of the case possess ample evidence that they are not only of unsound mind but are hopelessly insane. There is no doubt this effect will be produced on the mind by the perusal of passages in any one of a number of letters received from a patient labouring under partial, and as it proved, hopeless dementia. For example, in a letter

A Typical Letter.

written by a patient between thirty and forty years ago, the writer says: "I would that reason's voice were heard and that that voice could speak its notes to none but those whom conscience sways. Talk about principle! The man who attempts to be heard through the grates and tight lacings of a mad-house, must find hearers, not amongst the twaddling hypocrites of a close party clique, intent upon their own gains and dogmas, but to those who can clearly and impartially weigh evidence, deal with facts *pro* and *con*, and then give to the sufferer—the wronged —the benefit of all their doubt. 'Tis good sometimes to be plain, and I am quite sure that if the movements of this affair (admission into the asylum) were fully seen through, a more contemptible tissue of hypocrisy and cant could never be mixed up. The adage "oppression will make a wise man mad" is true. Talk about insanity! "Let my asylum be supported." I dare any man to keep my person in bondage. The present will come under the cognisance of Government the moment my liberty is obtained unless satisfactory reasons can be given. The whole of my life shall be spent in exposing by fair constitutional means the abominable system involved in the being shut up in such receptacles. I could wish my friends were aware of what it is to have one's liberty taken from one, one's circumstances destroyed, one's conscience attempted to be stifled, and the very reason biased by a system of pumping not unlike that of a steam-engine driving Truth into the minds of people. I do state, and that with perfect confidence, that the past has been a dishonourable state of things. Even supposing disease did exist, and an occasion for close confinement did arise, the very fact of being treated in an English asylum should have been enough to guarantee that it never became converted into a Bastille, nor made the means of coercing individual rights contrary to the acknowledged principles of life and the laws of the land. How to deal with the facts of the case I am at a loss to know! This much I do believe: the Archbishop of the Diocese must have been insulted, and the very clergy who have aimed at my restoration annoyed; and, if it be true that the mind suffers much from constant intermeddling with joys that belong to no other man, certain I am that the efforts of medical men to trace out the history of the Asylum, to gather them up, must be fraught with serious consequences to the nervous system, seeing that all is done without personal cognisance and regard to individual welfare. Never let it be said that a case has been made out for metaphysicians to hold up to posterity, nor that a precedent has been obtained for future doings of a similar kind."

**Legitimate Deductions.** Probably, if the whole correspondence of this patient were read, it would convince the most sceptical that the writer was insane, and perhaps some would suspect his sanity from certain obscure and irrelevant references in the latter part of this letter, from which only a very small proportion has been extracted. There are no doubt those who will say that "A Mental Physician" is entirely begging the question in the position he takes, that this patient was properly sent to and detained in an asylum. It may be stated, however, that it was one of those cases in regard to which there could not possibly be two opinions in the minds of those who resided in the same asylum. Such being the case in regard to a patient able to write sentences which would undoubtedly have the effect of inducing certain well-meaning but ill-informed persons to maintain that his confinement was improper and cruel, it is of great practical importance to make it clearly known to the laity that such letters are daily being written by persons totally unfit to be at large, and for whom and their friends an asylum is the greatest possible boon.

**Interfering Friends.** The injudicious interference of the friends of patients is not unfrequently a serious hindrance to their proper treatment and recovery. Let it not be supposed for a moment that from the time patients enter an asylum, the relatives should cease paying attention to them, or abstain from all notice of their complaints. Better, no doubt, to give ample opportunities to friends to visit patients, and to see for themselves the accommodation afforded them, notwithstanding the sometimes unreasonable and vexatious complaints which result, than to favour a system of secrecy, and to throw difficulties in the way of visitation. It is proceedings like the following, of which anyone having the charge of the insane has a right to complain. A gentleman was admitted into —— Asylum, labouring under mania. At times he was quiet, and even reasonable. A few days after admission his wife had an interview with him, and they were allowed to meet in the parlour of the institution. The lady, on leaving, allowed her husband to walk out with her, unobserved, to her lodgings. When his flight was discovered, several attendants were sent in search of him, and they proceeded to the lodgings. The lady having observed their approach, advised him to at once escape. Contrary to her expectations, he hesitated, but she led him to the back door and bade him betake himself to flight. This he did. Having visited an attorney, he took up his quarters at an inn, ordered wine, and began to write a letter. His wife, in accordance with an understanding between them, hastened to meet him there, not knowing that she was followed by the attendants. The patient assured her that such would be the case, but she asserted the contrary. When they appeared he asked them to be seated, offered them wine, and requested them to wait until he had finished his letter, adding (and this, we may observe by way of parenthesis, is of interest and importance), "if there had been only one of you I should not have come, but as there are three, I think I may as well." Being a powerful man of six feet, a smaller force would probably have ended in a severe struggle, in which the wife would have no doubt participated, physically or morally. The patient, having finished his letter, with deliberation rang the bell, paid for his wine, and quietly walked to the asylum with his wife, the attendants following at a respectful distance.

**After an Escape.** A subsequent episode illustrates in another way how uncalled for, after all, may be the interference of friends. When the patient passed into a quiet condition, the wish of his wife that she should take charge of him herself was granted, and he was formally discharged. On the day appointed the lady came for him, but then, to the astonishment of herself, and indeed of others, he positively refused to go. After he had left the room she desired to speak to him again, but he answered that he was smoking a cigar and could not see her. When we tried to persuade him to converse with her, he began to talk at random and was much excited. His wife was acutely pained, and probably realised more than she had done before, how injudiciously she had acted in inducing him to leave the asylum.

*(To be continued.)*

